# Empowering Underserved Communities in Southern Puerto Rico: A Formal Training Program in Community Health Promotion

**DOI:** 10.1007/s10900-024-01346-5

**Published:** 2024-04-04

**Authors:** Luisa Morales-Torres, David A. Vélez-Maldonado, Fernando J. Rosario-Maldonado, Jeannie M. Aguirre-Hernández, Jorge L. Motta-Pagán, Dorimar Rodríguez-Torruella, Eida Castro-Figueroa, Axel Ramos-Lucca, Elizabeth Rivera-Mateo, Melissa Marzán-Rodríguez, Julio Jiménez-Chávez

**Affiliations:** 1 Public Health Program, Ponce Health Sciences University, P.O. Box 7004, Ponce, 00732-7004 Puerto Rico; 2 School of Behavioral and Brain Sciences, Ponce Health Sciences University, P.O. Box 7004, Ponce, 00732-7004 Puerto Rico; 3 Ponce Research Institute, Ponce Health Sciences University, P.O. Box 7004, Ponce, 00732-7004 Puerto Rico

**Keywords:** Health promotion, Capacitation, CBPR, Implementation, Community leaders, CHW

## Abstract

Community health promotion offers a potential solution to persistent healthcare challenges, with community health workers playing a pivotal role. The Community Training Institute for Health Disparities (CTIHD) implemented a problem-solving curriculum in Community Health Promotion, integrating a competency-based learning model through two courses: Introduction to Community Health Promotion and Design of an Action Plan for the Promotion of Community Health. Each course comprised ten three-hour sessions, featuring pre/post-tests, evaluations, and a cognitive debriefing. Knowledge change was assessed using pre/post-test scores among 27 community leaders from southern Puerto Rico. Cohort 1 and Cohort 2 demonstrated an overall retention rate of 62.6% and 96.7%, respectively. Although differences in knowledge gained between cohorts and courses weren’t statistically significant, a trend toward increased knowledge was noted. Cohort 1 experienced a 22% knowledge increase in Course 1 and a 24% increase in Course 2. Cohort 2 demonstrated a 41% knowledge increase in Course 1 and a 25% increase in Course 2. The CTIHD’s Community Health Promotion Program has made significant strides in elevating awareness and knowledge, marking a positive step toward reducing health disparities and fostering healthier, empowered communities in southern Puerto Rico.

## Introduction

Addressing health disparities is a complex endeavor involving intricate factors that interact with and contribute to inequalities in health outcomes. Although some of these factors cannot be changed, others can be modified thereby making it imperative to focus on mitigating health disparities. These can arise from various risk factors and exposures, including tobacco use, alcohol consumption, environmental hazards, and genetic predisposition [1].

As a U.S. territory, Puerto Rico possesses a unique healthcare landscape shaped by historical, social, economic, and political influence. The island grapples with significant health disparities, leading to inequities in health outcomes among the Hispanic population [2]. These disparities encompass access to health care, disease prevalence, social determinants of health, and the enduring impact of natural disasters. Access to healthcare has emerged as a critical aspect of the health disparities in Puerto Rico [3]. The healthcare system on the island faces formidable challenges, including limited resources, shortage of healthcare providers, and unequal distribution of services [4]. High levels of poverty and unemployment have compounded these challenges, hindering many individuals from affording and accessing healthcare facilities [5]. This limited access to preventive care, screening, and early interventions contributes to elevated rates of chronic diseases and poorer health outcomes [6]. Chronic diseases such as diabetes, obesity, hypertension, and respiratory conditions are particularly prevalent in Puerto Rico [7]. Unhealthy diets, sedentary lifestyles, and limited access to nutritious foods further exacerbate the burden of chronic diseases [8]. Furthermore, the need for more resources for disease management and gaps in healthcare infrastructure hinder effective prevention and control efforts [9]. In response, stakeholders ranging from federal and local governments to private insurers, healthcare employers, researchers, and community advocates have explored community health promotion as a potential solution to address the persistent healthcare challenges [10, 11].

The importance of formal training and empowerment for community members is paramount in the effective implementation of Community Health Worker (CHW) based capacitation and training programs. Before individuals can assume the role of community health workers in tackling these challenges, they must receive comprehensive training to obtain the knowledge and skills to effectively promote health and empower others to make informed health decisions. A growing body of evidence supports the efficacy of (CHW) training programs in mitigating and managing chronic illnesses, as demonstrated by systematic reviews, by impacting patients with type II diabetes, asthma, coronary heart disease, stroke, and HIV [7, 12–19]. Moreover, such programs have benefitted vulnerable individuals at risk of developing chronic illnesses or cancer. Empowering community members to actively promote community health is crucial, as they possess an unparalleled understanding of their community’s unique health challenges and needs [20–22].

This article aims to highlight the implementation and evaluation of a problem-solving curriculum in Community Health Promotion, that integrates a competency-based learning model, to empower underserved Hispanic communities through a newly established Community Health Promotion Program by the Community Training Institute for Health Disparities (CTIHD). This innovative approach strives to address the distinctive health requirements and challenges encountered by socially disadvantaged Hispanic communities and fosters community involvement in identifying and resolving health concerns. By actively engaging community members in these processes, the program seeks to promote lasting changes and enable communities to achieve optimal health outcomes.

## Methods

### Curriculum Design

The curriculum design process was guided by a thorough examination of peer-reviewed literature, identifying existing programs that incorporate problem-based learning and competency-based learning models. This review provides valuable contextual insights for the expert panel responsible for shaping the curriculum. To conduct this comprehensive search, databases such as CINAHL, PubMed, PsycINFO, Social Sciences Full Text, and Google Scholar were used. Commonly used terms include "community health worker," "lay health worker," and "patient navigator" [23–27] which were used in conjunction with the concept of competencies [24, 28–30]. The CHW Curriculum establishes a set of six competencies to guide the development of its program curriculum. These competencies were designed to encompass health-related topics, focusing on knowledge acquisition, capacity building, organizational skills, teaching skills, interpersonal skills, and communication skills [30]. The formulation of these competencies was informed by a shared understanding of the skills and knowledge essential for CHWs to fulfill their community promoter’s role. However, the precise application of these competencies may vary depending on the CHW’s profession and context [31]

#### Program Structure

**Fig. 1:**
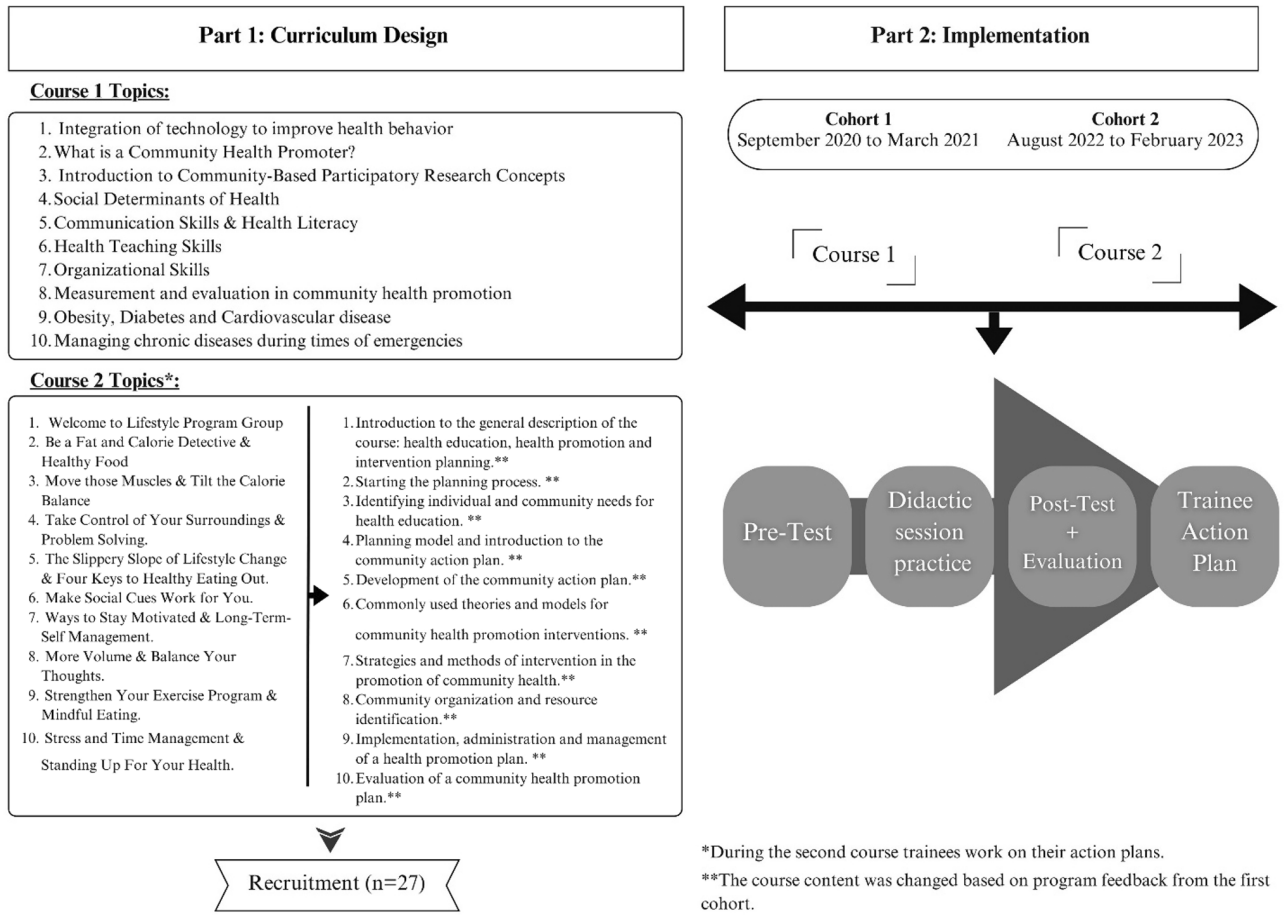
Diagram outlining the implementation of curriculum for Cohort 1 and Cohort 2 Adapted from the Community Health Worker Model

The curriculum was meticulously structured to encompass the vital components of community health promotion and divided into two distinct courses within the program (see Fig. 1). These courses, which are integral to the program’s success, are designed to equip participants with essential knowledge and skills. A competency matrix was developed meticulously to delineate specific learning objectives. Each intended learning outcome was intricately tied to the corresponding competency, encompassing the requisite knowledge, practical skills, and social acumen necessary for community health promotion. This framework serves as the foundation for the thematic outline of each course session. Introduction to the Community Health Promotion Course: This foundational course empowers participants with the essential tools required to become effective community health promoters. It delves into critical concepts, such as effective communication, motivational interviewing, organizational strategies, teaching methodologies, general principles of community-based participatory research, and an exploration of the social determinants of health. This comprehensive course focuses on the primary and secondary prevention of chronic diseases, including diabetes, cardiovascular disease, and obesity. Expert instructors possessing extensive community expertise facilitate this course, ensuring that participants receive the highest caliber of instruction.

Building on valuable feedback from participants in the inaugural cohort, enhancements were introduced in the second year of the program [31]. These refinements aimed to enhance the course’s effectiveness by placing greater emphasis on exploring and executing the essential steps required to devise and implement action plans to promote community health. Consequently, the course was aptly renamed "Design of an Action Plan for the Promotion of Community Health." Upon the successful completion of both courses, trainees are entrusted with the responsibility of conceiving and executing an outreach educational plan meticulously tailored to suit the unique needs of their respective communities.

#### Teaching and Learning Strategies Used

The aim of the program was to provide educational resources for students with at least a high school education. The approach used a range of teaching and evaluation techniques, such as peer education, teach-back, small-group presentations, role-play, didactic lessons, and individual assessments (see Fig. 1). These methods were incorporated to cater to various learning styles, ensuring that every student had an equal chance of success. Each syllabus was designed to be as straightforward and easy to understand as possible with a high school language level [31].

### Sample

**Fig. 2:**
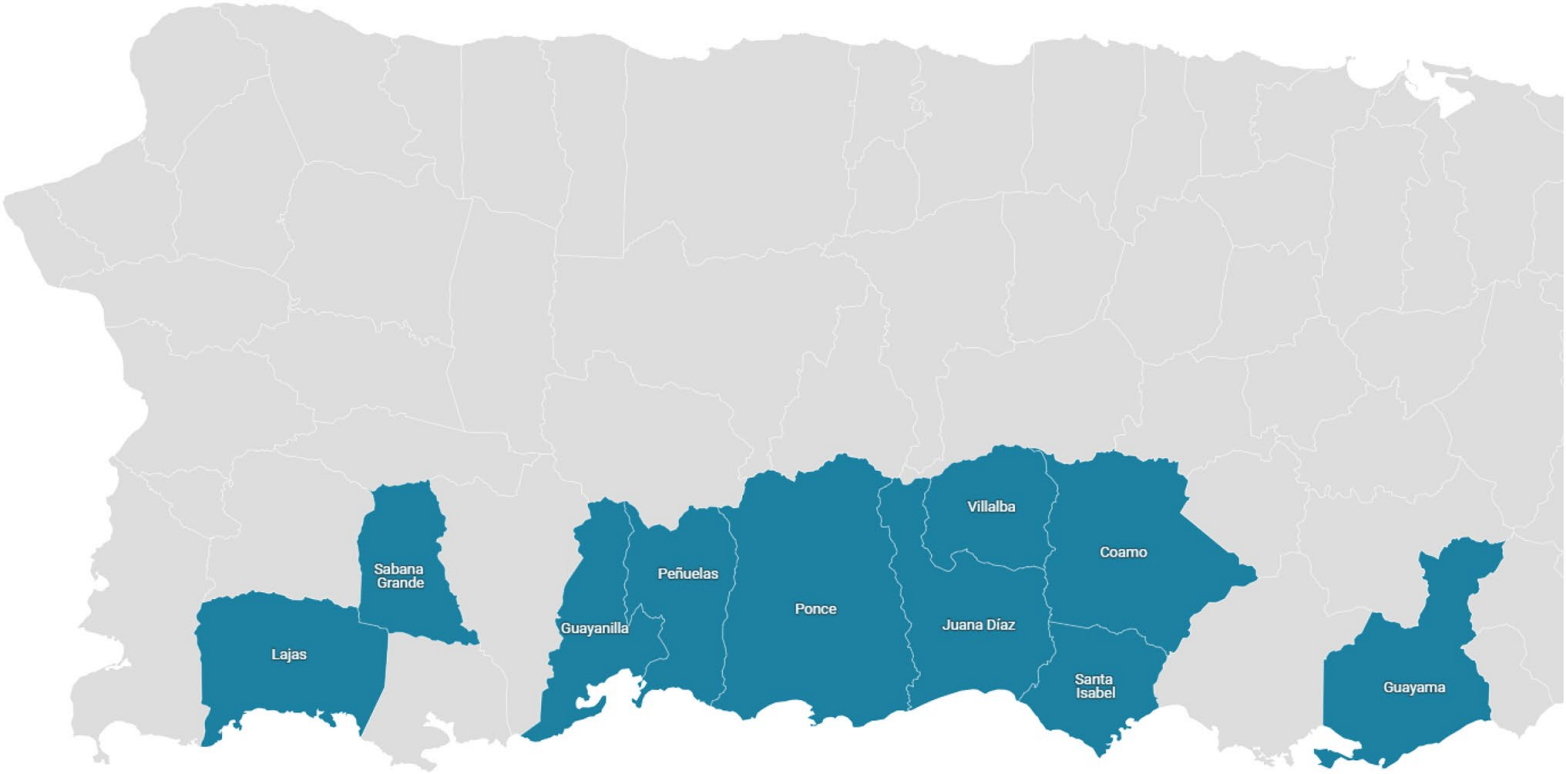
Municipalities impacted by the Community Health Promotion Program (Note. The figure presents the municipalities south of Puerto Rico impacted by the Community Health Program of the CTIHD. They are: Lajas, Sabana Grande, Guayanilla, Peñuelas, Ponce, Juan Díaz, Villalba, Coamo, Santa Isabel and Guayama.)

For the program’s participant selection, a rigorous procedure was employed that allowed us to enroll 27 community members residing in the southern region of Puerto Rico, utilizing a purposeful sampling approach (see Fig. 2 for the map area). The promotional strategies encompassed a diverse array of methods, including referrals from esteemed community leaders, direct invitations, distribution of recruitment flyers, and the use of social media channels. In the program’s inaugural year, a cohort of 12 participants was chosen, with an additional 15 participants recruited in the subsequent year.

**Fig. 3:**
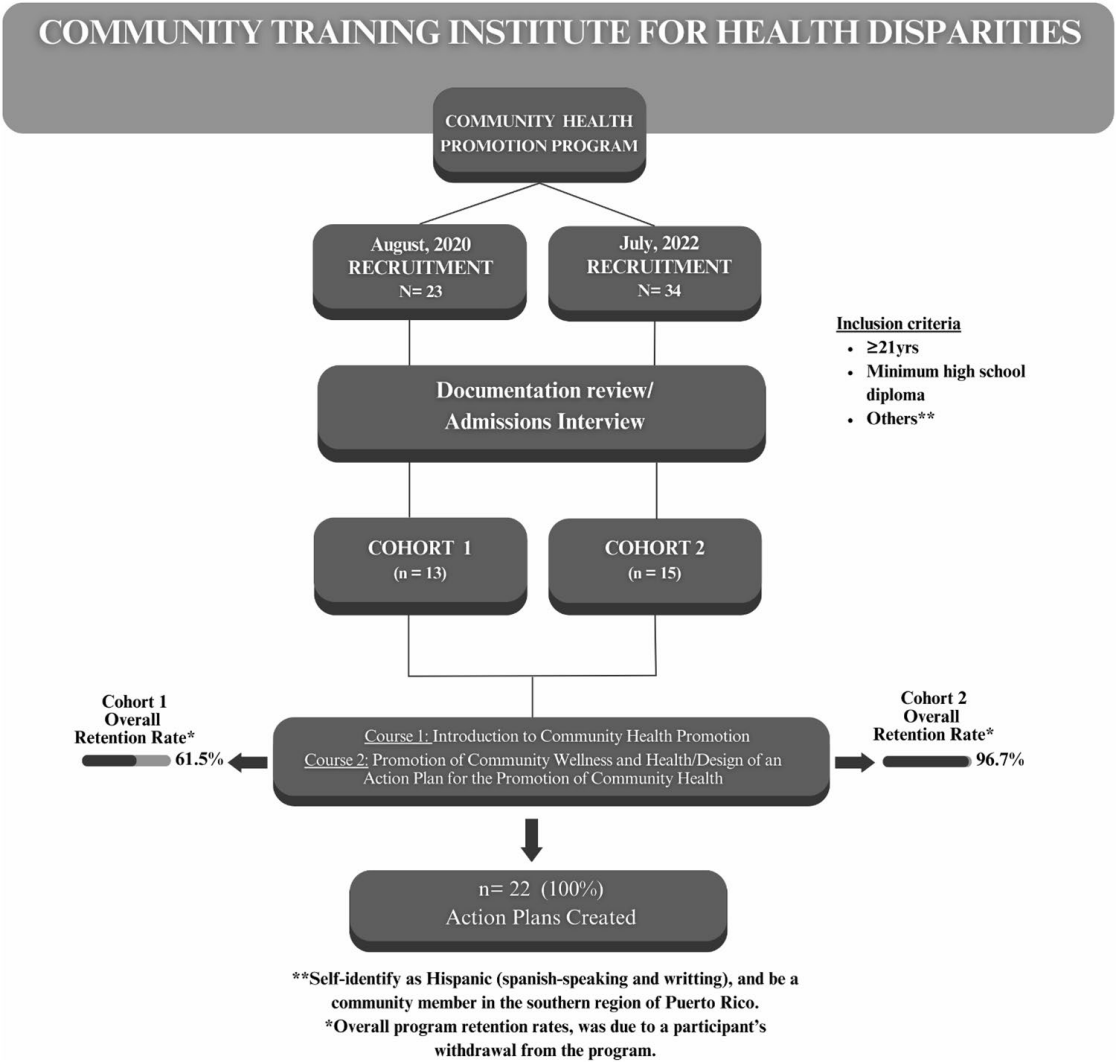
Program recruitment and implementation process

The fundamental aim of the participant selection process is to gauge potential candidates’ interest and experience of community engagement and character. This comprehensive assessment involved a multifaceted evaluation encompassing interviews, scrutiny of resumes, consideration of letters of recommendation, examination of personal statements, and site visits to the candidate’s respective communities. Participants who satisfactorily completed the requisite documentation and demonstrated their suitability during the interview phase were invited to participate in the program. The interview process itself was a collaborative endeavor involving the potential candidate, a community partner from the CTIHD, the program leader, and an administrative assistant. The assessments were meticulously conducted and evaluated using a standardized rubric. In the first year, 19 of the 23 applicants underwent interviews, ultimately leading to the selection of 12 individuals (63%) for Cohort 1. In the second year, from a pool of 24 applicants, 23 were interviewed and 15 (65%) were selected for cohort 2 (see Fig. 3). The reasons for non-selection included incomplete documentation, scheduling constraints, and instances in which candidates chose not to proceed with the application process. It is worth noting that the project received ethical approval from the Institutional Review Board (IRB) of Ponce Health Sciences University, with protocol number 2107068687.

### Measures

#### Retention Rate

As of August 2020, the program’s initial cohort witnessed the enrollment of twelve individuals, whereas in August 2022, Cohort 2 welcomed fifteen (15) participants. A prerequisite for program completion was that attendees were required to actively participate in a minimum of nine out of the ten scheduled sessions. Furthermore, attendees were expected to provide evidence of having reviewed any missed sessions due to their absence, thus ensuring their full engagement and commitment to the program.

#### Program Evaluation

The Community Health Promotion Program utilized a multi-method approach, comprising of cognitive debriefing through semi-structured questions (i.e., qualitative), a sociodemographic survey, a course evaluation questionnaire, and pre- and post-tests (i.e., quantitative).

##### Quantitative Evaluation

*Sociodemographic Survey* Sociodemographic information was collected from the trainee group at the start of the program through a questionnaire survey. The objective was to create a comprehensive profile of the participants, encompassing their origin, age, gender, sexual identity or orientation, marital status, household composition, level of education, place of residence, and affiliation with various types of organizations.

*Change in Knowledge and Satisfaction* The evaluation process encompassed two vital aspects: gauging the enhancement of participants’ knowledge and assessing their overall satisfaction with the program. Comprehensive data were collected for each participant, including both pre- and post-test quizzes, along with satisfaction surveys for each module. The quizzes were designed to have a maximum attainable score of 10, and the percentage of correct answers was calculated meticulously for each module. To ensure clarity and consistency of language, ten multiple-choice questions for each module were thoughtfully crafted by the respective lecturers, and these questions were approved by the component leader.

*Course Evaluations* After finishing each training program, the trainee filled out a questionnaire consisting of six domains: Course Structure and Organization, Course Learning Experiences, Instructor Effectiveness, Course Evaluation Criteria, Virtual Tools Used and Technologies, and General Satisfaction.

##### Qualitative Evaluation

*Cognitive Debriefings* After each implementation period, the participants underwent cognitive debriefing led by a trained interviewer. The questions for the debriefing were in a semi-structured format, and each group session lasted between 30 and 60 min, depending on the level of participation. The session was conducted with eight to twelve participants to gather additional details that could not be gathered in the evaluation during program implementation. Suggestions from the cognitive debriefing sessions were reviewed and incorporated into subsequent implementation periods to improve the quality of the program.

### Analysis

#### Quantitative Analysis

##### Change in Knowledge and Satisfaction

The principal outcome variable for further analysis was derived from the post-test scores. A paired t-test with a two-tailed approach was used to compare pre-test and post-test scores. The Wilcoxon rank test was used to evaluate the change in knowledge using the pre/post test scores. Statistical significance was set at p < 0.05. Data was presented as the mean values and standard deviations. SPSS version 28 was used to conduct this statistical analysis, ensuring a robust and reliable assessment of knowledge improvement and participant satisfaction.

#### Qualitative Analysis

##### Cognitive Debriefings

The session covered a range of questions related to the program’s structure, course order, educational and practical activities, schedule, professors, materials provided, and the selection criteria. Additionally, it addressed questions about the suitability of the course content for community members; the process of developing an action plan for Community Health Promotion; the strategies, resources, or factors that participants found helpful in managing challenges; their preparedness to develop and implement an action plan; their readiness to educate the community; the program’s contribution to community problems or settings; and overall satisfaction with the program’s activities. Content was analyzed, depending on the responses they were categorized in the previously mentioned topic questions.

## Results

The results of this study provide valuable insights into the sociodemographic characteristics of participants in the Community Health Promotion Program of the CTIHD (Community Training Institute for Health Disparities) for the period spanning from 2020 to 2023.

For cohort 1 of the 12 recruited participants, nine completed the first course and eight completed the second (75% retention rate for course 1 and 67% retention rate for course 2), for an overall program retention rate of 62.6%. In contrast, for cohort 2 of the 15 recruited participants, 15 completed the first course and 14 completed the second (100% retention rate for course 1 and 93.3% retention rate for course 2), for an overall program retention rate of 96.7% (see Fig. 3).

In Table 1, most of the participants in the program were female (90.9%), while only a small percentage were male (9.0%). Participants’ ages ranged from 24 to 64 years, with a mean age of 47 years. Participants exhibited diverse marital statuses, with married individuals (45.5%) forming the largest group, followed by single/never married individuals (14.8%), and those living with a partner (22.7%). The distribution of participants across income categories revealed a range of socioeconomic backgrounds. While a substantial proportion had annual incomes below $10,000 (22.7%), others reported higher incomes, including those earning $100,000 or more (13.6%). A significant number of participants had attained a bachelor’s degree (44.4%), while others had completed high school (11.1%) or achieved advanced degrees, including master’s (18.5%) and doctoral degrees (7.4%). The participants came from various municipalities in southern Puerto Rico, with Ponce (44.4%) having the largest representation.

**Table 1:** Participant demographics in the CTIHD Community Health Promotion Program (2020–2023)

	Overall N (%)	Cohort 1 n (%)	Cohort 2 n (%)
Sex			
Male	2 (9.0)	1 (12.5)	1 (7.1)
Female	20 (90.9)	7 (87.5)	13 (92.9)
Total	22 (100)	8 (100)	14 (100)
Age			
Mean (years) ± SD	47 ± 12.4	40 ± 14.3	51 ± 9.4
Range (min–max)	24–64	24–60	33–64
Marital status			
Single/never married	4 (18.2)	1 (12.5)	3 (21.4)
Married	10 (45.5)	2 (25.0)	8 (57.1)
Divorced	2 (9.1)	1 (12.5)	1 (7.1)
Living with a partner	5 (22.7)	4 (50.0)	1 (7.1)
Widowed	1 (4.5)	–	1 (7.1)
Prefer not to answer	3 (13.6)	–	
Annual income			
< 10,000	5 (22.7)	2 (25.0)	1 (7.1)
10,000–14,999	4 (18.2)	–	4 (28.6)
15,000–24,999	2 (9.1)	2 (25.0)	–
25,000–34,999	1 (4.5)	–	1(7.1)
35,999–49,999	5 (22.7)	2 (25.0)	3 (21.4)
50,999–74,999	4 (18.2)	–	4 (28.6)
100,000 or more	3 (13.6)	2 (25.0)	1 (7.1)
Prefer not to answer	3 (13.6)	–	–
Educational level			
Completed high school	3 (13.6)	1 (12.5)	–
Associate degree	4 (18.2)	1 (12.5)	2 (14.3)
Technical degree	1 (4.5)	1 (12.5)	–
Bachelor’s degree	12 (44.5)	3 (37.5)	7 (50.0)
Master’s degree	5 (22.7)	2 (25.0)	3 (21.4)
Doctorate’s degree	2 (9.1)	–	2 (14.3)
Municipality			
Coamo	3 (13.6)	1 (12.5)	1 (7.1)
Guayama	1 (4.5)	1 (12.5)	–
Guayanilla	1 (4.5)	–	1 (7.1)
Juana Díaz	1 (4.5)	–	1 (7.1)
Lajas	1 (4.5)	1 (12.5)	–
Peñuelas	3 (13.6)	–	3 (21.4)
Ponce	9 (40.9)	3 (37.5)	6 (42.9)
Sábana Grande	1 (4.5)	1 (12.5)	–
Santa Isabel	2 (9.1)	1 (12.5)	1 (7.1)
Villalba	2 (9.1)	–	1 (7.1)

Out of the 27 participants, a total of 22 successfully completed the Community Health Promotion curriculum

### Program Effectiveness

The evaluation of the Community Health Promotion Program’s effectiveness was conducted by comparing pre- and post-test scores for different courses and cohorts.

#### Course 1 Cohort 1

**Fig. 4:**
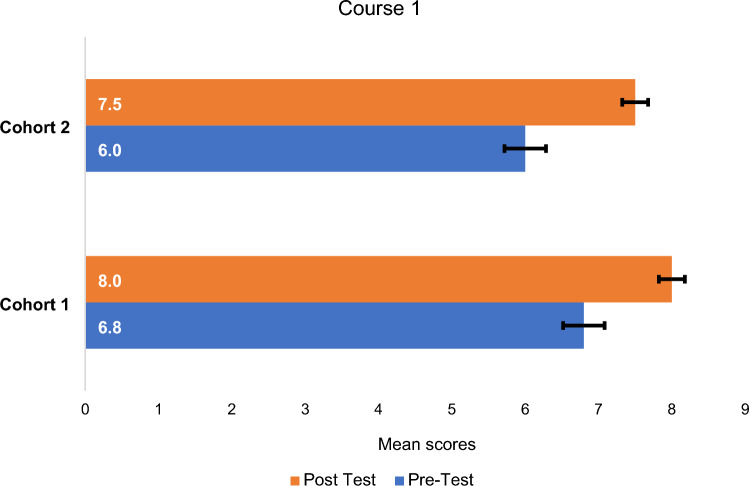
Average participants result of pre/post-test for course 1 for cohort 1 (n = 8) and cohort 2 (n = 14)

Table 2, the results indicated a positive change in knowledge, with an overall 22% improvement in scores after Course 1. Notably, Sessions 4 and 5 demonstrated substantial knowledge gains of 24% and 58%, respectively. Figure 4 shows the mean pre- and post-test scores of each cohort for Course 1.

**Table 2:** Results of the paired t test measuring the difference in knowledge before and after Course 1 participation in Cohort 1 participants

Session	n of course 1 participants	Mean pre-test score	Mean post-test score	Difference	% Change	P-value
1	10	7.4	8.7	1.27	17	0.3696
2	10	7.4	8.7	1.30	18	0.2601
3	10	4.8	5.5	0.70	15	0.4315
4	10	7.1	8.8	1.70	24	0.0326
5	9	4.4	7.0	2.56	58	0.0184
6	8	5.1	8.4	3.25	63	0.0475
7	7	8.4	9.3	0.86	10	0.1789
8	8	6.0	7.0	1.00	17	0.2071
9	9	8.1	7.7	0.44	− 5	0.3984
10	9	8.9	9.4	0.56	6	0.1765
Overall		6.8	8.0	1.40	22	0.2120

#### Course 2 Cohort 1

**Fig. 5:**
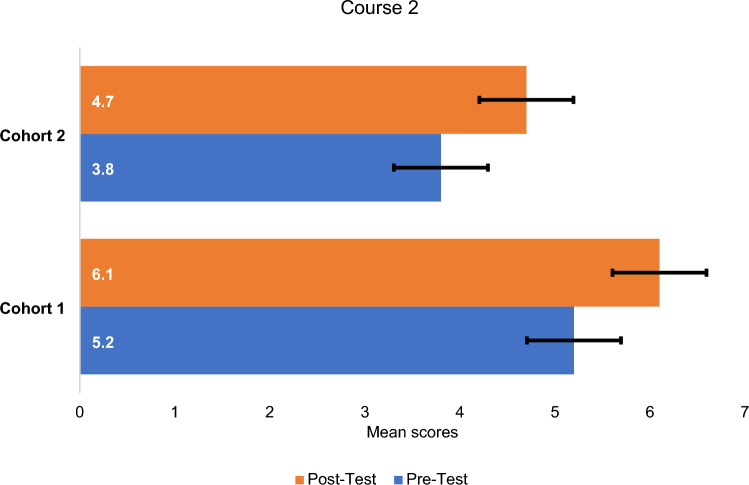
Average participants result of pre/post-test for course 2 for cohort 1(n = 8) and cohort 2 (n = 14)

Course 2 for Cohort 1 also exhibited knowledge improvements, with an overall 24% increase in the scores (see Table 2). Session 3 demonstrated the most significant improvement (37%), suggesting that certain course topics had a particularly strong impact on the participants’ understanding of community health promotion. Figure 5 shows the mean pre- and post-test scores of each cohort for Course 2. Upon close examination of Course 2 sessions, it was found that session 10 exhibited an 86% difference in knowledge compared to the remaining sessions in the course (see Table 3).

**Table 3:** Results of the paired t test measuring the difference in knowledge before and after Course 2 participation in Cohort 1 participants

Session	n of course 2 participants	Mean pre-test score	Mean post-test score	Difference	% Change	P-value
1	8	4.0	5.0	1.00	25	0.0670
2	8	6.6	7.4	0.75	11	0.2273
3	8	3.8	5.1	1.38	37	0.0388
4	8	5.9	6.1	0.25	4	0.7590
5	7	4.3	5.0	0.71	17	0.3767
6	7	8.1	7.8	0.31	− 4	0.3446
7	7	5.7	6.3	0.57	10	0.4536
8	8	4.8	7.0	2.17	45	0.0481
9	8	6.0	6.7	0.67	11	0.4472
10	8	2.3	4.3	2.00	86	0.0803
Overall		5.2	6.1	0.98	24	0.2842

#### Course 2 Cohort 2

On Table 4, Cohort 2 participants in course 2 showed a noteworthy 25% overall improvement in knowledge, with session 1 indicating a 10% increase. The subsequent sessions showed consistent improvements, with session 5 demonstrating a substantial 31% increase. These findings suggest that Course 2 has a positive impact on knowledge across both cohorts, see also Fig. 5.

**Table 4:** Results of the paired t test measuring the difference in knowledge before and after Course 2 participation in Cohort 2 participants

Session	n of course 2 participants	Mean pre-test score	Mean post-test score	Difference	% Change	P-value
1	14	4.5	4.9	0.47	10	0.0276
2	14	4.5	4.7	0.21	5	0.3265
3	14	4.0	4.9	0.86	21	0.0001
4	14	3.9	4.7	0.86	22	0.0025
5	14	3.7	4.9	1.14	31	0.0000
6	14	3.9	4.5	0.64	17	0.0246
7	14	4.0	4.3	0.29	7	0.5196
8	14	2.7	4.1	1.43	53	0.0037
9	14	3.6	5.0	1.43	40	0.0000
10	14	3.1	4.5	1.36	43	0.0003
Overall		3.8	4.7	0.87	25	0.0905

### Cognitive Debriefing

After the completion of all courses, a cognitive debriefing was conducted at the end of the academic year to gather feedback from the community trainees from each cohort. The feedback was organized based on predefined themes and subthemes, as shown in Table 5.

**Table 5:** End of program cognitive debriefing—Community Health Promotion Program

Evaluated criteria	Responses
General Recommendations for Program: structure, courses order, educational-practical activity, schedules, professors, Materials provided and selection criteria	• The sessions should be more interactive and be in person; otherwise, I feel satisfied • I would have liked the hybrid (online) and in-person format. It would be great if both options were offered • The second course should have been a continuation of course 1 so that it was consistent with the development and implementation of educational plans • The schedule is comfortable and effective • I liked the format of course 1 • These spaces for discussion and learning are open to diverse groups • The first course seemed very diverse in subject matter; I would’ve liked that the second to be equally diverse and not focused on a “coaching” topic, focusing on other social and environmental issues, in addition to chronic diseases • In a biopsychosocial approach, I liked the variation in teaching resources, as it made Course 1 more dynamic • I would like to begin working with communities from the beginning of the program • Course 2 could have had schedule flexibility because of its extensive content. I felt that the implementation process was enriching as a practical activity • A community leader who lacks that experience might benefit from adapting the language for individuals with less expertise
Process of developing an action plan for health promotion	• It was not previously discussed how an educational plan was developed during the sessions but at the end of the course. The discussion element would have been beneficial in understanding this elaboration • I liked the feedback process for drafting my educational plan • It was difficult to prepare the educational plan in terms of structure to determine how to write it. I would have liked more direction • My recommendation for the design of the educational plan is that we need a "hands on" session where we can maybe give each other support creating those plans right where we live
Strategies, resources, or factors that participant found helpful in managing challenges	• Because I work in the health field, it became a challenge to follow through with the sessions because of (personal) time. The strategy I used was to select a topic that I dominated because the time factor was against me • The program’s personnel assistance was a valuable resource • My visual disability prevented me from seeing the presentations; therefore, the assistance of the team and program with the provision of a laptop was extremely beneficial to me • Support from the program team motivated me to complete and implement my educational plan. The follow-up received helped me successfully implement my plan • The tools that you used from the videos of the recording of the class were essential for me • Having the academic calendar available from the beginning of the program helped you a lot to schedule. And to be able to participate in the sessions
Readiness to educate the community	• I feel prepared to educate a community under certain conditions and bring education to the rehabilitation process. I feel prepared to expand on these topics • With the training received, I feel prepared to educate others, since I had the opportunity to do so by offering terrorist training • The program gave me a preamble to address other issues in the community, where I directed a project where I developed an educational plan to educate different organizations and identities that offer services to the elderly population in my main workplace • I was able to adapt this to the format learned in the program
Program’s contribution to community problems or settings	• The program opens the door to recent and relevant topics. It is a good step to bring information closer to communities and the leadership that represents them. It is essential to integrate more community leaders into the process. Like this program has done • This program can be the beginning of many projects, and health topics cover a lot. Therefore, more people are required to start this process • This program serves as an excellent basis for many projects • I believe that this program opened many doors for us, letting us know that we can reach the community and that we can work in collaboration and alliances. And that we can offer our services with credibility
Overall satisfaction with the program’s activities	• I am very satisfied and grateful for this opportunity • The program met my expectations • I feel very happy for the support and am grateful for the process and challenges overcome • In general, it has been diverse, we have had different themes. I also believe that we have been able to create a community among ourselves
Additional comments	• The second course is more comprehensive regarding strategies that can be implemented in different communities. Consider addressing other topics or strategies that may be more effective when focusing on the implementation of an educational plan or intervention • Course 2 offers sufficient tools to implement any educational plan or strategy on any public health topic that is of interest to communities

### Course Evaluations

At the conclusion of each program course, trainees were required to evaluate their learning experience. As shown in Table 6, the results of these evaluations indicated that the domains evaluated for program courses were generally of a high standard. In particular, the program’s overall percentage scores were highest in Course Structure and Organization (96.9%), Instructor Effectiveness (96.9%), and Tools Used and Technologies (96.9%). Furthermore, the overall General Satisfaction rate was 95.3%.

**Table 6:** Overall program course evaluation by criteria—Community Health Promotion Program

Evaluated criteria	Cohort 1 (%)	Cohort 2 (%)	Program overall (%)
Course structure and organization	93.8	100	96.9
Course learning experiences	93.8	96.7	95.3
Instructor effectiveness	93.8	100	96.9
Course evaluation criteria	88.2	100	90.8
Virtual tools used and technologies	93.8	100	96.9
General satisfaction	93.8	96.7	95.3

### Program Impact

Twenty-two community members completed the program and developed health education plans. Health fairs, educational presentations (in-person or virtual), and educational booths were strategies used by trainees to promote healthy behaviors to reduce the risk of developing chronic diseases and comorbidities in their targeted population. Forty-five percent (45%) of the trainees implemented their educational interventions in 10 communities on topics such as diabetes, cancer, mental health, nutrition, sex education, violence among young adults and obesity, to name a few. The different educational interventions impacted 224 individuals (from children to adults).

## Discussion

The findings presented in this study shed light on the sociodemographic characteristics of participants in the Community Health Promotion Program of the CTIHD (Community Training Institute for Health Disparities) spanning from 2020 to 2023. Additionally, the evaluation of program effectiveness through pre- and post-test scores for different courses and cohorts offers valuable insights into the impact on participants’ knowledge of community health promotion.

The sociodemographic profile of the program participants revealed a diverse group in terms of age, marital status, income, educational level, and geographic location. This diversity is a testament to the program’s ability to engage a wide range of individuals from various backgrounds, contributing to a more inclusive and comprehensive approach to community health promotion. Importantly, the program demonstrated its effectiveness in enhancing the participants’ knowledge of community health promotion. Across different courses and cohorts, participants showed significant improvements in their understanding of the key concepts and practices related to community health promotion. Noteworthy knowledge gains were observed in specific sessions, indicating that certain topics resonated strongly with the participants and had a substantial impact on their learning.

Considering the challenges encountered during the course, it was not noted as a barrier for trainees to fulfill the certification requirements of the program and institute. The overall satisfaction of trainees from both cohorts is evidenced by the retention rate and comments made during cognitive debriefing, such as: "The program gave me a foundation to address other issues in the community," "I am very grateful for the support and challenges I overcame during the process," and "The program has been diverse and we have covered various themes. I am also proud of the community we have built among ourselves." Regarding the academic structure of the program, trainees from the first cohort made valuable contributions and recommendations that led to changes in the general structure and thematic content of Course 2, the balance between theoretical and practical activities, and the schedule of the program. One trainee from the first cohort expressed, "The second course should have been a continuation of Course 1 for it to be consistent with the development and implementation of educational plans." These changes were implemented in the second cohort of trainees, who also had more in-person interactions with instructors, more practical activities, and additional days to better understand the course materials.

Cohort 1 trainees attended their courses remotely (i.e., online) due to the COVID-19 pandemic. This posed potential barriers and limitations for effective communication between trainees and instructors [32, 33], leading to a high possibility of burnout and exhaustion. The social and emotional toll posed by COVID-19 [34], has also made some trainees emotionally vulnerable, interfering with their ability to concentrate and participate in the courses. To address these challenges, the institute’s administrative team assigned trainees an academic advisor, academic professor, and licensed clinical psychologist who evaluated the situation to provide psychosocial and mental health services as needed.

Moreover, during the first year of implementation, the program’s geographic location, Puerto Rico, was affected by a large earthquake in January 2020 [35]. In addition, a low level of literacy and limited access to technology among some trainees turned out to be additional barriers to academic engagement. This made equitable participation impossible compared to other trainees. To address this disparity, the research team provided basic training on the technologies to be used and the required computer equipment. However, this became even more complex as some trainees lived in rural and remote areas where the internet signal was weak and unstable, or they did not have access to a Wi-Fi connection. In response, the conferences were recorded to ensure that the trainees could watch them at another time, if needed.

These findings underscore the importance of the program as a valuable tool for addressing health disparities among the underserved populations in southern Puerto Rico. By equipping participants with increased awareness and knowledge in community health promotion, the program has the potential to empower individuals and communities to make informed decisions and take proactive steps towards improving their health and well-being. The results of this study should inform future program development and refinement, with a focus on building upon the observed strengths and addressing any areas where further improvements can be made. As the program continues its mission to serve and uplift underserved communities, ongoing evaluation and adaptation will be crucial to maximize its impact.

The implementation of a problem-based curriculum for community health promotion, integrated with a competency-based learning model, is important for capacitating, supporting, and empowering community members, particularly those hailing from socially disadvantaged Hispanic communities. These communities often grapple with the profound impact of chronic illnesses, health disparities, and health inequities, making this approach a crucial and timely endeavor. One of the key strengths of the program is its ability to address the pressing needs of underserved populations who bear the heavy burden of chronic illnesses, health disparities, and health inequities. By amalgamating problem-based learning with competency-based models, the program equips individuals with practical skills and knowledge that is directly applicable to the specific challenges faced by their communities. This practical orientation ensures that participants are not only well informed but also capable of taking meaningful actions to bring about positive health changes.

Moreover, the program’s specific focus on socially disadvantaged Hispanic communities is paramount in addressing the persistent health disparities experienced by these populations. Health disparities encompassing differences in health outcomes and access to healthcare services have plagued these communities for generations. Consequently, there is an urgent need for targeted interventions that cater to unique circumstances and requirements. In this context, the Community Health Promotion Program emerges as a beacon of hope, offering individuals from these communities a tangible pathway to advocate for transformative and equitable change.

## Conclusions

The Community Health Promotion Program of the CTIHD has made significant strides in advancing knowledge and awareness of community health promotion among its participants. This progress marks a positive step toward reducing health disparities and fostering healthier and more empowered communities in southern Puerto Rico. As the program continues to evolve and expand its reach, it holds the promise of creating lasting positive impacts on the health and well-being of these communities. While this study has limitations, such as its sample size and potential for selection bias, it also possesses several strengths, including its innovative program design, diverse participant representation, and the positive knowledge gains observed. Further research and program refinement can build upon these strengths to maximize the program’s effectiveness.
